# SA-responsive transcription factor GbMYB36 promotes flavonol accumulation in *Ginkgo biloba*

**DOI:** 10.48130/FR-2023-0019

**Published:** 2023-08-10

**Authors:** Jinkai Lu, Peixi Tong, Yuan Xu, Sian Liu, Biao Jin, Fuliang Cao, Li Wang

**Affiliations:** 1 College of Horticulture and Landscape Architecture, Yangzhou University, Yangzhou 225009, China; 2 Co-Innovation Center for Sustainable Forestry in Southern China, Nanjing Forestry University, Nanjing 210037, China

**Keywords:** Salicylic acid, *Ginkgo biloba*, Flavonoid biosynthesis, *GbF3'H*, *GbMYB36*

## Abstract

Flavonoids are abundant secondary metabolites in *Ginkgo biloba* and have a wide range of medicinal values. Salicylic acid (SA) can induce flavonoid accumulation in plants, but the detailed regulatory mechanism remains unclear. Here, we established the optimal media for ginkgo callus induction and subculture, and found that exogenous SA greatly increased the content of flavonol, including quercetin, kaempferol, isorhamnetin. Transcriptome changes in SA-treated calli showed that most structural genes involved in flavonoid biosynthesis were upregulated. Particularly, overexpression of *GbF3′H* in ginkgo calli significantly increased the content of flavonol, suggesting the vital role of *GbF3′H* in flavonoid biosynthesis. We further identified that a R2R3-MYB, GbMYB36, were significantly upregulated in SA treated calli. Transient overexpression and a LUC assay indicate that *GbMYB36* act as an activator, and improve flavonoid biosynthesis through regulating the expression of *GbF3′H*. Our findings provide insight into the molecular basis of SA-induced flavonoid biosynthesis in ginkgo.

## Introduction

Flavonoids are vital secondary metabolic products that play crucial roles in the immune and defense responses of plants. Flavonoids can be subdivided into diverse families, including flavonols, flavones, isoflavones, anthocyanidins, flavanones, flavanols, and chalcones^[1−3]^. Flavonoids are synthesized *via* the phenylpropanoid pathway, of which several key structural genes have been identified^[4]^. During early steps of the flavonoid biosynthesis pathway, phenylalanine ammonia-lyase, 4-coumarate-CoA ligase, and cinnamic acid 4-hydroxylase genes are involved in the deamination of phenylalanine to 4-coumaroyl-CoA^[5,6]^. Subsequently, chalcone synthase (CHS) and chalcone isomerase (CHI) catalyze the synthesis of naringenin, which serves as a central precursor for the production of most downstream flavonoids^[7,8]^. The pathway diverges from naringenin into several side branches, each of which is enzymatically converted to a different class of flavonoids by the action of downstream biosynthetic genes, such as flavonol synthase (FLS) and flavanone 3′-hydroxylase (F3′H) genes^[7,9]^.

There is growing evidence that transcriptional levels of these structural genes are predominantly regulated by several transcription factors (TFs) directly binding to their promoters, including WRKY, MYB, bHLH, WD40, and bZIP proteins^[10,11]^. TFs act independently or in combination with other TFs from the same or different families to regulate the expression of target genes^[12]^. Generally, the regulation of flavonoid biosynthesis involves the combined action of the MYB–bHLH–WD40 (MBW) complex^[13]^. In the MBW complex, MYBs have been the most comprehensively researched TFs and are considered the most specific and prominent regulators^[14]^. MYBs, as the activators of flavonoid biosynthesis, have been widely reported in plants, including *AtMYB11*, *AtMYB12*, and *AtMYB111* in *Arabidopsis thaliana*^[15]^, *MdMYB9*, *MdMYB11*, and *MdMYBPA1* in apple^[16,17]^, *PbMYB10b* and *PbMYB9* in pear^[18]^, and *RrMYB5* and *RrMYB10* in *Rosa rugosa*^[19]^.

The biosynthesis of flavonoids is regulated by several internal and external factors, of which plant hormones function as the pivotal regulators^[20]^. Salicylic acid (SA) is an effective hormone elicitor that induces the production of flavonoids in plants^[21]^. Recent studies have revealed that SA treatment can induce the transcription of flavonoid biosynthesis genes, resulting in the accumulation of high levels of flavonoid compounds in *Arabidopsis thaliana*^[22]^, tea^[20]^, and poplar^[23]^. Nevertheless, the regulation mechanism of SA-induced flavonoid accumulation in plants remains unknown.

*Ginkgo biloba* L. is an economically and biologically important tree species that contains several important secondary compounds, such as terpenoids, flavonoids, alkylphenol acids, and ginkgolides^[9,24,25]^. Because of its considerable pharmacological value, *G. biloba* has been subjected to extensive medicinal and chemical investigations. *G. biloba* leaf extract (GBE) is one of the best-selling herbal preparations due to its ability to treat cardiovascular diseases, dementia, and tinnitus^[26,27]^. Multitudinous GBE-based drugs or food supplements are available worldwide, and flavonoids are considered the primary pharmacological components in GBE^[28,29]^. Although several key structural genes including *FLS*, *CHS*, *CHI*, and *F3′H* encoding multiple enzymes have been identified in *G. biloba*, how to enhance flavonoid biosynthesis has remained a consistent research hotspot in recent years^[30,31]^.

*In vitro* culture of plants has evolved as a promising biotechnology approach for the commercial production of target compounds^[21,32]^. Therefore, *in vitro* tissue culture of ginkgo is an effective method to increase the yield of flavonoids^[33]^. However, a series of biological and biotechnological problems in ginkgo culture, including low callus induction ratios, callus browning, and low yield of target compounds, still need to be overcome^[33,34]^. In this study, we established the optimal induction and subculture media for ginkgo calli, and found that SA can greatly increase the flavonol glycoside contents. On this basis, we identified GbMYB36 as the activator and regulated the key structural gene *GbF3′H*, suggesting the essential role in SA-induced flavonol accumulation.

## Materials and methods

### Plant material and tissue culture methodology

Healthy and fresh *G. biloba* leaves were collected from the ginkgo plantation in Yangzhou University, Jiangsu Province, China. The leaves were rinsed with tap water for 2 h. Leaf explants were sterilized using 70% (v/v) alcohol for 5 min, followed by washing with aseptic water three times for 5 min each time. Subsequently, the leaves were aseptically cut into 0.5 cm × 0.5 cm sections and immediately cultured in ten different media to screen for the optimal induction medium (Supplementary Table S1). The subculture was conducted on 1-month-old calli. Three different concentrations of three browning inhibitors, namely, activated carbon (AC), polyvinylpyrrolidone (PVP), and vitamin C (VC), were added to the subculture medium. The browning inhibitor was not added to the control group.

### Exogenous SA treatment

After 20 d of subculture, the calli were removed and transferred to a subculture medium supplemented with SA (10 μM). After 5 d of subculture with SA, several calli were collected from the medium for analysis of total flavonoids and flavonol glycosides contents. Other calli were preserved in liquid nitrogen and stored at −80 °C for transcriptome analyses. For the treatment of ginkgo seedlings, two-month-old seedlings were selected and then treated with SA (1 mM) and ABT (0.5 mM). The leaves were harvested 24 h after the SA and ABT treatment.

### Measurement of chlorophyll and polyphenol oxidase activity

Frozen calli (0.1 g) were crushed in liquid nitrogen. The methods to extract chlorophyll (Chl) from calli powder followed those of Lu et al.^[31]^. Chl content were determined using ultraviolet (UV)–visible spectrophotometry and the absorbances at 665 nm and 649 nm were measured. The total Chl content was determined as the sum of the contents of Chl a and Chl b. The Chl a content was calculated as (12.19 A_665_ − 3.45 A_649_)/20. The Chl b content was calculated as (21.99 A_649_ − 5.32 A_655_)/20.

The polyphenol oxidase (PPO) activity was measured following the manufacturer’s instructions (PPO Activity Detection Kit; Solarbio, China). The absorbance was monitored at 410 nm on a UV–visible spectrophotometer and the activity of the enzyme was expressed as unit·mg^−1^ protein (U·mg^−1^) of the homogenate.

### Extraction and quantification of flavonol glycosides and total flavonoids

Dried ginkgo calli were pulverized into powder to extract flavonol (quercetin, kaempferol and isorhamnetin). Dry calli (0.1 g) were extracted with 2 mL of 70% ethanol (v/v). Subsequently, the supernatant was evaporated using a rotary evaporator and dissolved in 200 µL of 25% (v/v) HCl–methanol for 10 min by ultrasound. The solution was centrifuged for 30 s and transferred to a 10 mL chemical oxygen demand tube with a Teflon liner, followed by heating at 85 °C for 30 min and cooling at 4 °C for 10 min. The samples were mixed with 200 µL of methanol and collected by centrifugation. The obtained samples were filtered using an organic membrane with a pore diameter of 0.22 µm before performing high-performance liquid chromatography (HPLC) analyses. Total flavonol glycosides content was calculated by multiplying total quercetin, kaempferol and isorhamnetin by a factor of 2.51^[30]^. In addition, the total flavonoid content was determined using a plant flavonoid kit (Suzhou Comin Biotechnology Co., Suzhou, China).

### Transcriptome sequencing and differentially expressed genes analysis

Samples for transcriptome analysis were collected from the calli after 5 d of SA treatment. Approximately 1 µg of RNA per sample was sequenced, followed by construction of six sequencing libraries (SA treatment and control groups). Low-quality, adaptor, and poly-N sequences in the raw data were filtered out. The filtered reads were mapped to the ginkgo genome (http://gigadb.org/dataset/100613) using TopHat2. Fragments per kilobase of transcript per million mapped reads (FPKM) was used to quantify the gene expression. Differentially expressed genes (DEGs) between the control and SA groups were identified using the DESeq2 package in R (version 1.16.1), based on *p*-values < 0.05 and fold change ≥ 2. Kyoto Encyclopedia of Genes and Genomes (KEGG) pathway analyses of DEGs were conducted using the clusterProfiler package in R according to the KEGG database (www.genome.jp/kegg). The raw data were deposited in the Genome Sequence Archive of the National Genomics Data Center under the accession number CRA005679.

### Bioinformatic analysis

Genomic DNA sequences of 2,000 bp upstream of *GbF3′H* were submitted to PlantCARE to identify the putative *cis*-element regulatory DNA elements (http://bioinformatics.psb.ugent.be/webtools/plantcare/html/). Other associated protein sequences from multiple species (*Triadica sebifera*, *Oryza sativa*, *Arabidopsis thaliana*, *Vitis vinifera*, *Juglans sigillata*, *Prunus persica*, *Narcissus tazetta*, *Prunus cerasifera*, *Morus alba*, *Taxus chinensis*, and *Pinus taeda*) were downloaded from National Center for Biotechnology Information (NCBI) (www.ncbi.nlm.nih.gov). A phylogenetic tree was generated from the protein sequences of F3′H by MEGA7 using the neighbor-joining method. The conserved motifs displayed were predicted using the online version of MEME^[35]^.

### Quantitative reverse-transcription PCR analysis

Total RNA from callus and leaf samples was extracted using the Plant RNA Rapid Extraction Kit (Vazyme Biotech Co., Nanjing, China). Elimination of genomic DNA contamination and first strand cDNA synthesis were performed using the PrimeScript RT Reagent Kit with gDNA Eraser (Vazyme Biotech Co.). Quantitative reverse-transcription (qRT)-PCR was conducted using SYBR qPCR Master Mix reagents (Vazyme Biotech Co.). The expression of the reference gene actin was used to quantify the relative expression levels. Biological triplicates were used for expression assays of each gene. Supplemental Table S3 lists primer sequences used for qRT-PCR analysis.

### *Ginkgo* calli transformation

The full-length coding sequences (CDSs) (without termination codons) of these genes were amplified from the cDNA of leaves. Next, the PCR fragments were ligated to the intermediate vector pMD-19T. The plasmid was digested using the restriction enzyme *BamHI*, and the fragments were inserted into the pRI 101-AN expression vector driven by the cauliflower mosaic virus (CaMV) 35S promoter. For ginkgo callus transformation, the recombinant plasmids were transformed into the ginkgo calli using the *Agrobacterium tumefaciens* GV3101-mediated method. Calli infected with *Agrobacterium* containing the empty vector (EV) were used as a control. The primers used are listed in Supplemental Table S3.

### Transient overexpression assays in tobacco leaves

Transient overexpression assay was conducted in young leaves of 20-day-old seedlings of *Nicotiana tabacum*. The CDS of *GbMYB36* was cloned into pRI 101-AN vector and then transformed into *Agrobacterium* strain. Cultures were grown to an OD_600_ of 0.8, then resuspended in 30 mL of infiltration buffer. The infection solution was incubated for 3–4 h at 25 °C in the dark and then used for transient transformation experiments.

*Agrobacterium* cultures containing GbMYB36 was injected into the back of tobacco leaves. The seedlings were placed in the dark for 16 h, then transferred to a culture chamber under long-day conditions (23 °C/16 h light; 18 °C/8 h dark). After 7 d, leaf samples were collected for determination of flavonoid contents. EV infiltrations (pRI 101-AN) were used as negative controls.

### Dual-luciferase assay

The promoter region upstream from the start codon of *GbF3′H* (1.5 kb) was amplified from ginkgo genomic DNA and inserted into the multiple cloning site of vector pGreenII0800LUC to construct luciferase reporter plasmid. The effector and reporter constructs were separately transformed into *A. tumefaciens* strain GV3101 (pSoup). Then, *Agrobacterium* cells containing recombinant plasmids were co-infected into *N. benthamiana* leaves. After 3 d of infiltration, 0.5 mM D-luciferin potassium salt was used as a luminescent substrate. Subsequently, the leaves of the transformed tobacco were collected and sprayed with diluted luminescent substrate. LUC luminescence was captured using an imaging system for living plants (Tanon-5200, China).

### Statistical analyses

All physiological data (PPO activity, Chl, flavonol, total flavonol glycosides, and total flavonoids) are expressed as means ± standard deviations. Student’s two-sided *t*-tests and analysis of variance (ANOVA) with *post hoc* tests were used to assess the statistical significance of differences between treatment and control conditions. *P*-values < 0.05 and < 0.01 were considered to indicate statistical significance.

## Results

### Establishment of callus induction and subculture media for ginkgo

During *in vitro* culture, the appearance of calli in explants is an indicator of growth. The type, concentration, and combination of plant growth regulators in the medium can affect the induction of calli. We used leaves of ginkgo as explants and applied 2,4-dichlorophenoxyacetic acid, 1-naphthylacetic acid (NAA), and kinetin in the culture media (M1–M9) to induce the calli (Supplemental Table S1). Based on the callus induction rate, we observed that the medium containing 4.0 mg·L^−1^ NAA and 2.0 mg·L^−1^ kinetin (M6) was the most effective for induction (Supplemental Table S2). Thus, the M6 medium was used in subsequent experiments.

**Figure 1:**
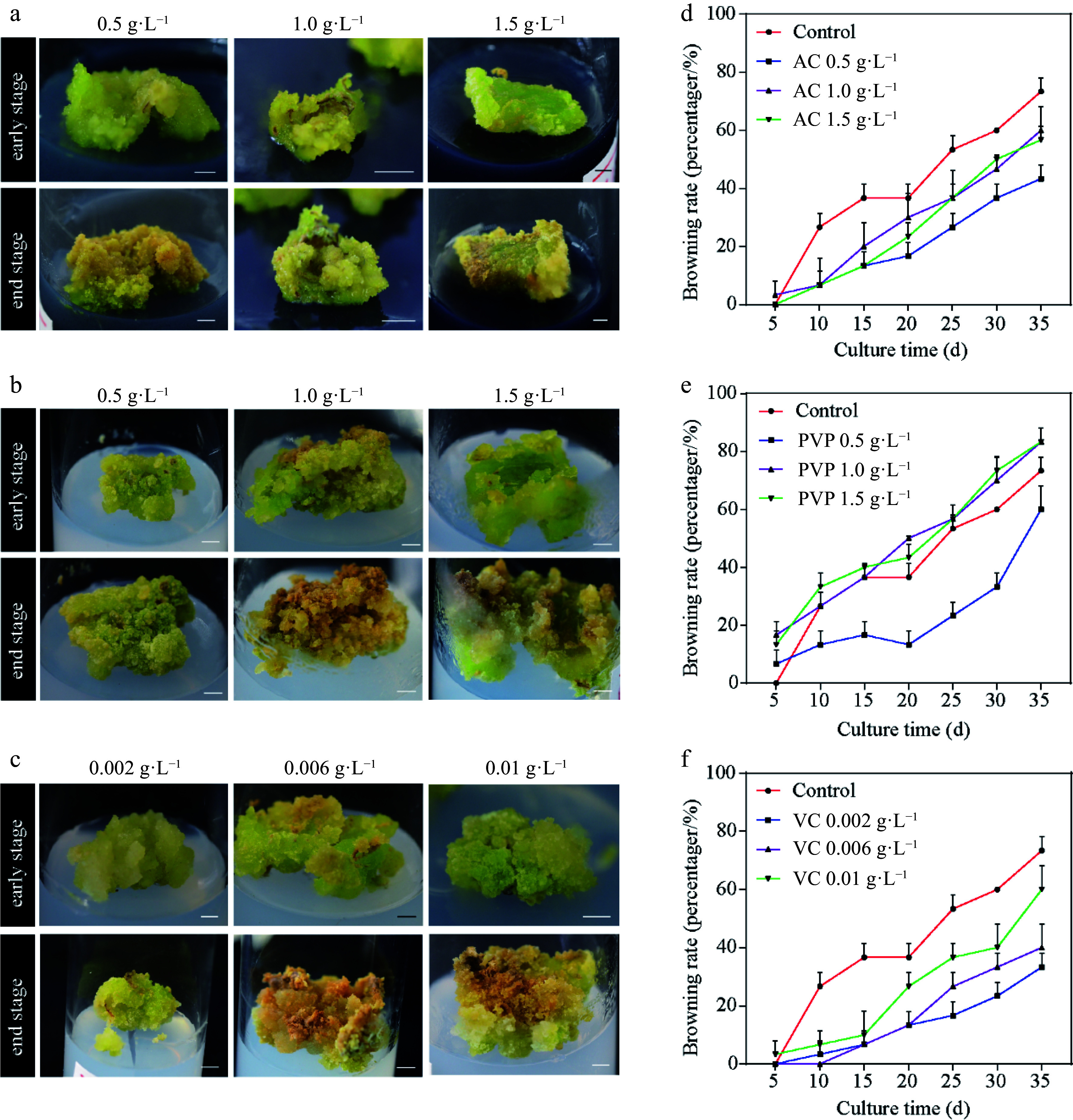
Callus induction from *Ginkgo biloba* leaves and browning rates. The morphology of calli cultured on the induction medium supplemented with different concentrations of browning inhibitors. (a) 0.5, 1.0, and 1.5 g·L^−1^ activated carbon (AC). (b) 0.5, 1.0, and 1.5 g·L^−1^ polyvinylpyrrolidone (PVP). (c) 0.002, 0.006, and 0.01 g·L^−1^ vitamin C (VC). (d)–(f) Effects of different concentrations of AC, PVP, and VC on the browning rate of ginkgo calli. Scale bars = 5 mm.

**Figure 2:**
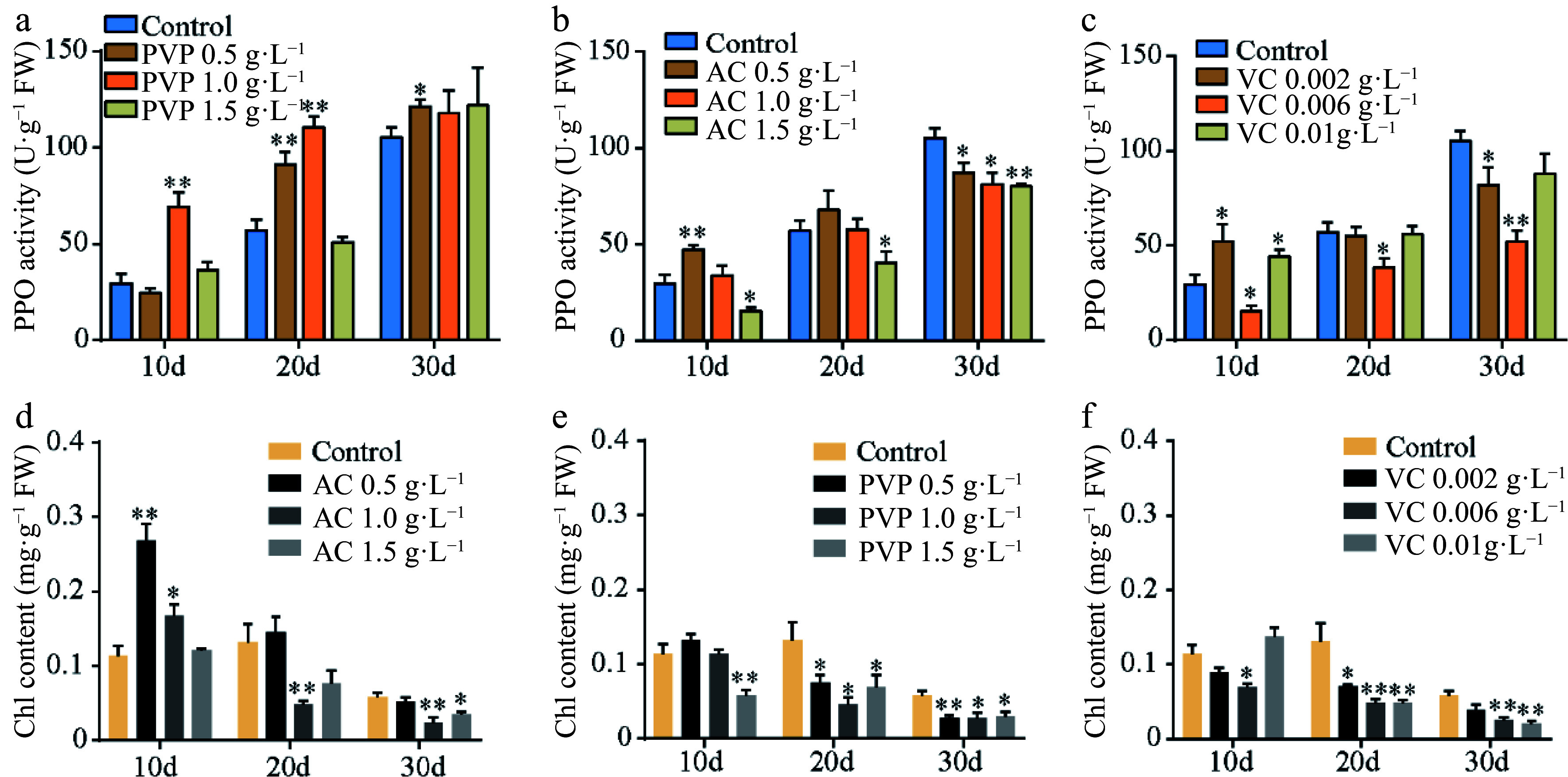
Polyphenol oxidase (PPO) activity and chlorophyll contents. (a)–(c) The PPO activity and (d)–(f) chlorophyll content under different concentrations of AC, PVP, and VC treatments. Statistical significance: * *p* < 0.05; ** *p* < 0.01.

Callus browning affects the subculturing of ginkgo calli; therefore, we further screened the subculture media. As shown in Fig. 1, the callus was green on medium supplemented with 0.5 g·L^−1^ PVP and 0.002 g·L^−1^ VC; however, it turned pale yellow and friable in structure at increased concentrations (Fig. 1a–c). In addition, treatment with low VC concentrations (0.002 g·L^−1^ and 0.006 g·L^−1^) exerted a higher anti-browning effect than AC or PVP (Fig. 1d–f). Callus browning is attributed to polyphenolic compounds that are oxidized by PPO when the explants are damaged^[36]^. Significant differences in the PPO activity were observed following treatment with different concentrations of AC, PVP, and VC (Fig. 2a–c). At the early stage of callus culture, all treatments showed a lower PPO activity level. However, the PPO activity increased in different treatments with time. In particular, 0.006 g·L^−1^ VC treatment showed the lowest mean PPO activity among all treatments. We further observed continuous reduction in the contents of chlorophyll (Chl) with the extension in the culture time (Fig. 2d–f). However, 0.5 g·L^−1^ AC, 0.5 g·L^−1^ PVP, and 0.002 g·L^−1^ VC effectively induced the accumulation of Chl; in particular, 0.002 g·L^−1^ VC showed effective anti-browning activity and was optimal for the growth of ginkgo calli. In addition, the calli showed good growth after 20 d of subculture.

### Effect of SA on the biosynthesis and accumulation of flavonol

**Figure 3:**
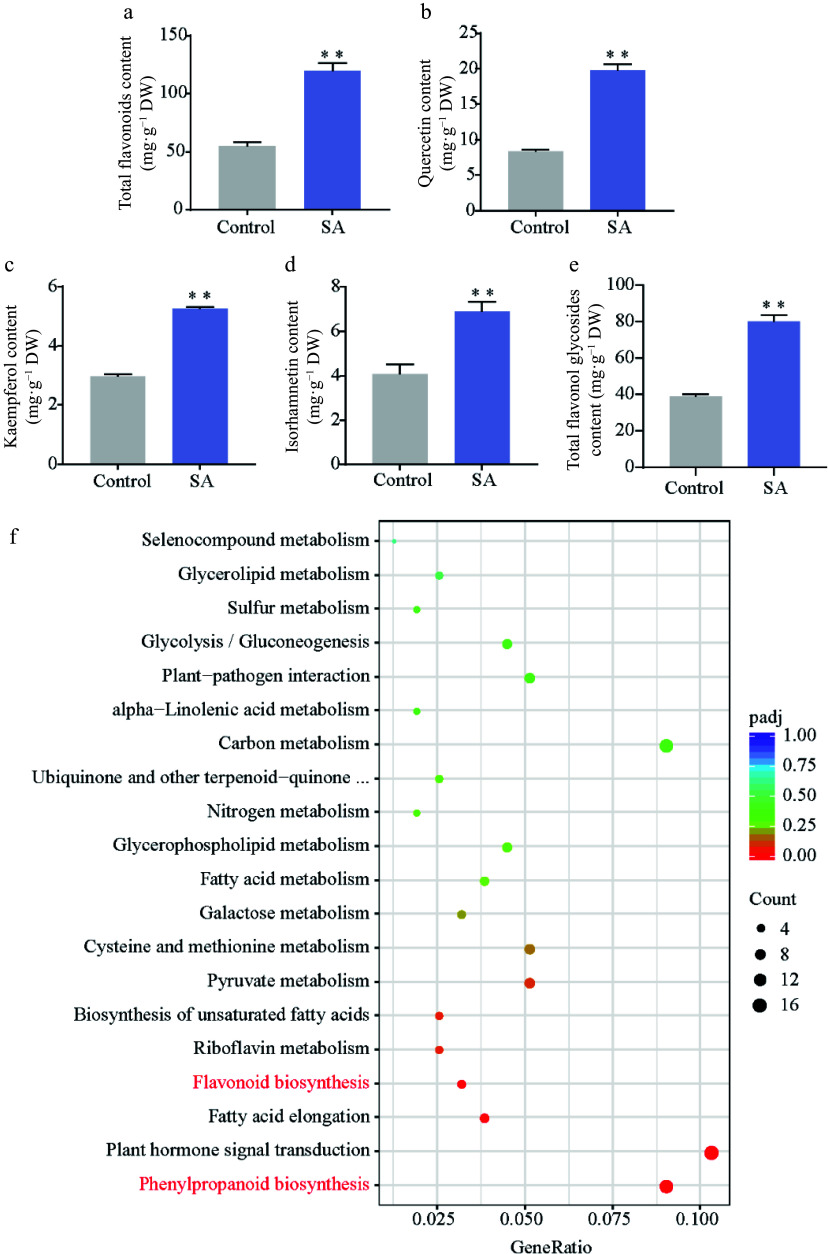
Effect of SA on flavonoid content. (a) Total flavonoid content. (b)–(e) Content of flavonol in SA-treated calli and control. (f) KEGG pathway enrichment of DEGs responses to SA treatment. Statistical significance: * *p* < 0.05; ** *p* < 0.01.

SA is an important abiotic elicitor for the synthesis of secondary metabolites. Based on the optimal subculture medium, we added 10 μM SA and found that the total flavonoid content increased by approximately 117.9% with an average of 119.7 mg·g^−1^ in SA-treated calli compared to the control (54.9 mg·g^−1^) (Fig. 3a). Furthermore, HPLC analyses showed that the content of quercetin, kaempferol, isorhamnetin, and total flavonol glycosides increased by 69% to 135% in SA-treated calli (Supplemental Fig. S1; Fig. 3b–e).

**Figure 4:**
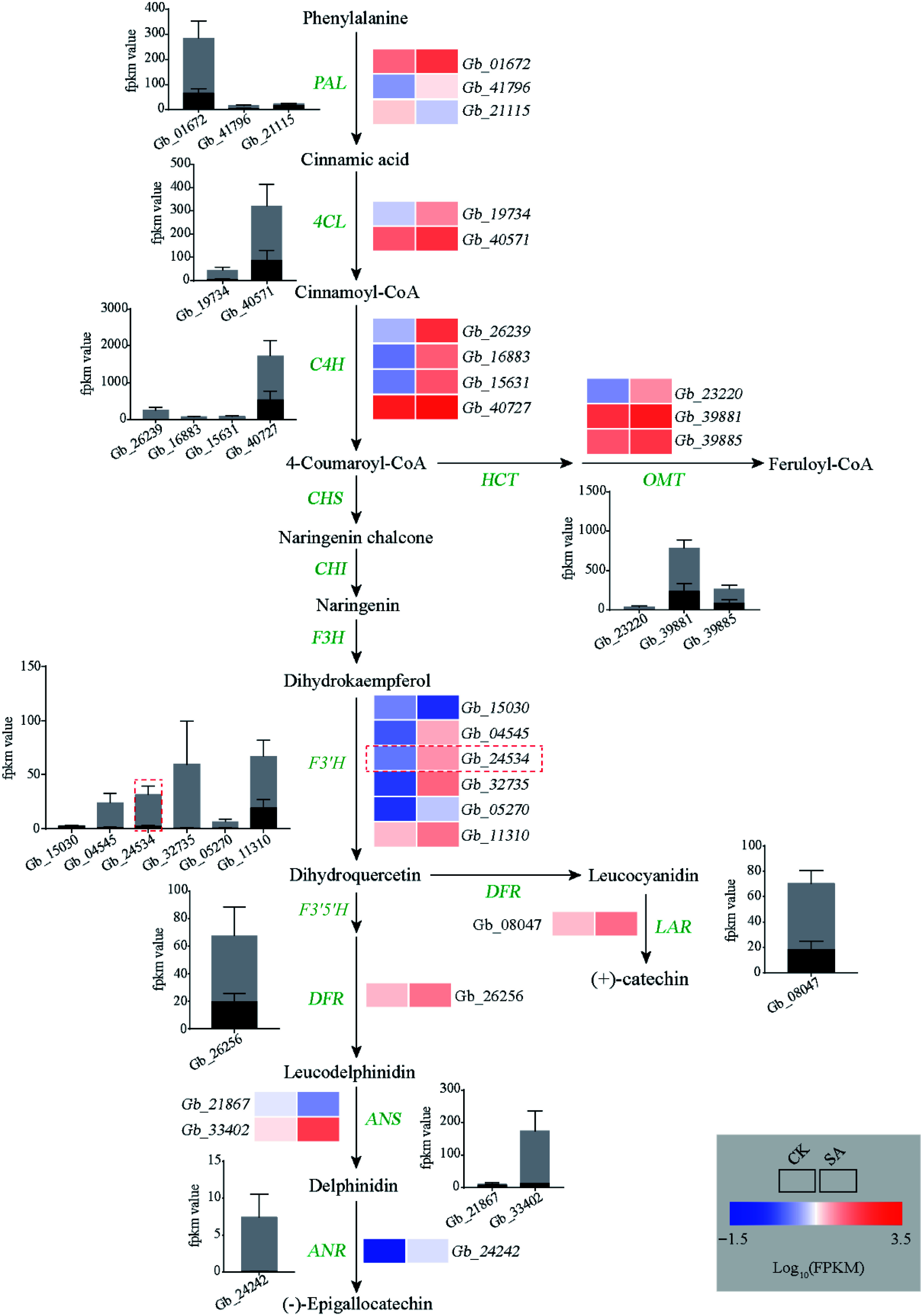
Analyses of flavonoid biosynthesis pathway. Heat map showing changes in the transcripts of genes involved in flavonoid metabolism. Rectangles marked with red (upregulation) and blue (downregulation) backgrounds represent the average log_10_ (FPKM) value of each pathway gene according to the color scale. The black and gray columns represent average expression levels of genes in control and SA treatment samples, respectively.

To explore the mechanism of SA-induced flavonoid biosynthesis in the ginkgo calli, transcriptome analysis was performed on SA-treated calli and untreated calli. A total of 2,991 DEGs were identified between the control and SA treatment, comprising 1,770 upregulated genes and 1,221 downregulated genes in SA-treated calli (Supplemental Fig. S2). The KEGG enrichment analysis revealed that phenylpropanoid biosynthesis pathway and flavonoid biosynthesis pathway were significantly enriched among these DEGs (Fig. 3f). We next focused on the phenylpropanoid biosynthesis pathway and found almost all structural genes (20/23) were upregulated in the SA-treated calli. Among these, the expression of phenylalanine ammonia-lyase (*PAL*), O-methyltransferase (*OMT*), *F3′H*, and dihydroflavonol 4-reductase (*DFR*) was significantly upregulated (approximately 2- to 97-fold). In particular, we identified that the transcription levels of five *F3′H* genes (5/6) participated in the production of dihydroquercetin, a precursor of quercetin biosynthesis, was significantly upregulated following the SA treatment (Fig. 4).

### Overexpression of *GbF3'H* in ginkgo resulted in increased flavonol contents

We found that four vital *F3′H* genes (*Gb_04545*, *Gb_24534*, *Gb_32735* and *Gb_11310*) were significantly increased and highly expressed after SA treatment. We then measured the expression levels of these genes using qPCR, and found that the expression of these genes significantly increased after SA treatment, with *Gb_24534* experiencing the greatest up-regulation in expression. These results suggest that *Gb_24534* may play a vital role in SA-induced flavonoid synthesis. (Supplemental Fig. S3). Next, we cloned complete CDS sequence of *GbF3′H* (*Gb_24534*). Promoter sequences up to 2,000 bp upstream from the translation start site of *Gb_24534* were scanned by the PlantCare program to identify *cis*-acting regulatory elements. Several different *cis*-elements participated in response to plant hormones were observed in the *GbF3′H* promoter sequence (Supplemental Fig. S4a). Phylogenetic analysis showed that *GbF3′H* was closely related to *F3′H* in gymnosperms including *Taxus chinensis* and *Pinus taeda*. The other *F3′H* genes in angiosperms were relatively distantly related to *GbF3′H*. Protein sequence analysis indicated *F3′H* genes have many conserved motifs sharing among gymnosperm to angiosperms (Supplemental Fig. S4b).

**Figure 5:**
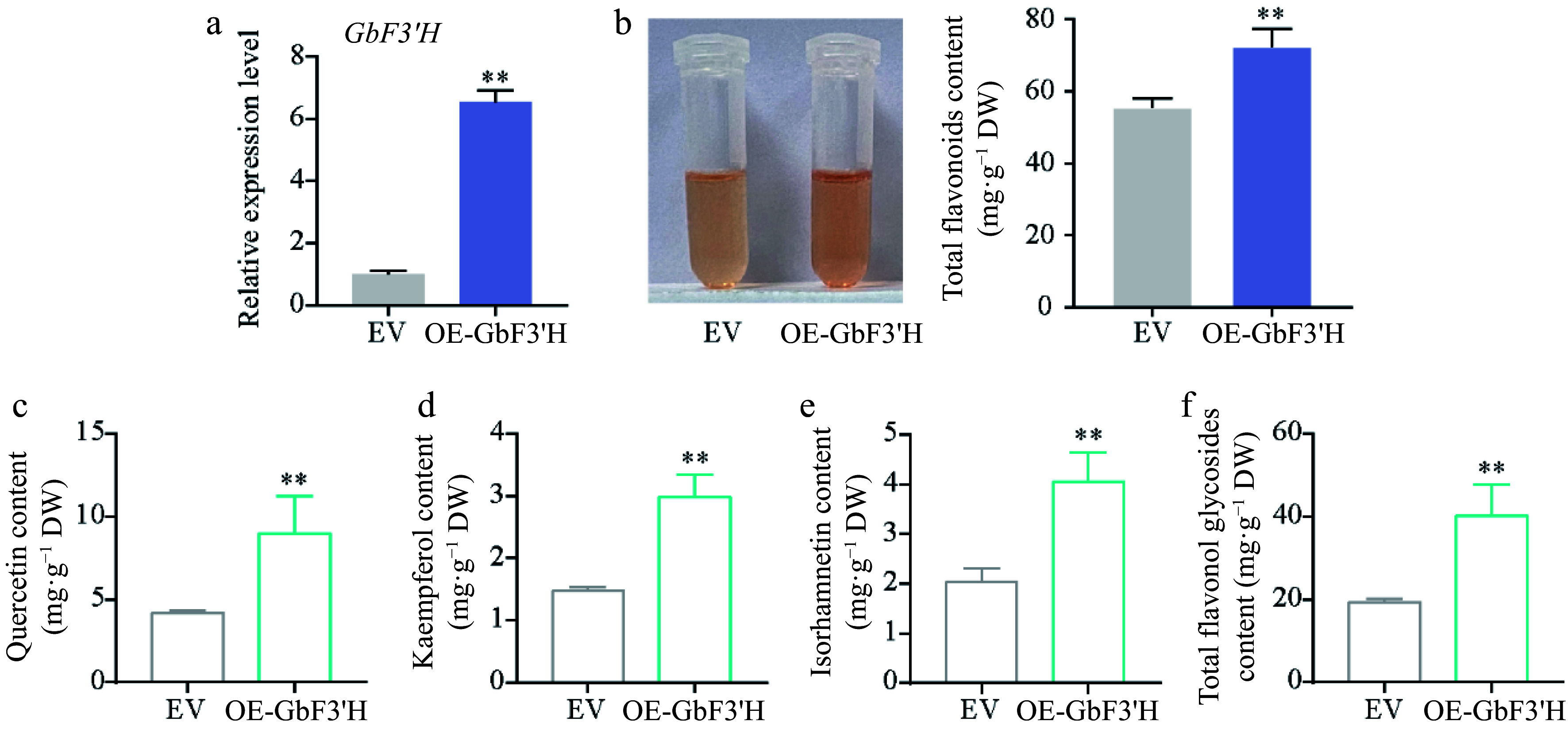
Functional analysis of *GbF3′H* involved in flavonoid biosynthesis in ginkgo. (a) qRT-PCR confirmation of *GbF3′H*-overexpressing transgenic ginkgo calli. (b) Total flavonoid contents in the EV and OE-GbF3′H calli. (c)–(f) HPLC analyses of flavonol in the EV and OE-GbF3′H calli. Statistical significance: * *p* < 0.05; ** *p* < 0.01.

To explore the functions of *GbF3′H*, the gene was genetically transformed into the ginkgo calli. Compared with the empty vector-transformed (EV) calli, the expression levels of *GbF3′H* in the *GbF3′H*-transformed (OE-GbF3′H) calli were significantly increased (Fig. 5a). Meanwhile, compared to the EV calli, the contents of total flavonoids in OE-GbF3′H calli were significantly increased (Fig. 5b). Similarly, HPLC analyses showed that the contents of quercetin, kaempferol, isorhamnetin, and total flavonol glycosides also significantly increased in OE-GbF3′H calli (Supplemental Fig. S1; Fig. 5c–f). These results indicated that *GbF3′H* is the key structure gene that regulates flavonoid biosynthesis in *G. biloba*.

### Identification of candidate transcription factors involved in the flavonoid biosynthesis pathway

**Figure 6:**
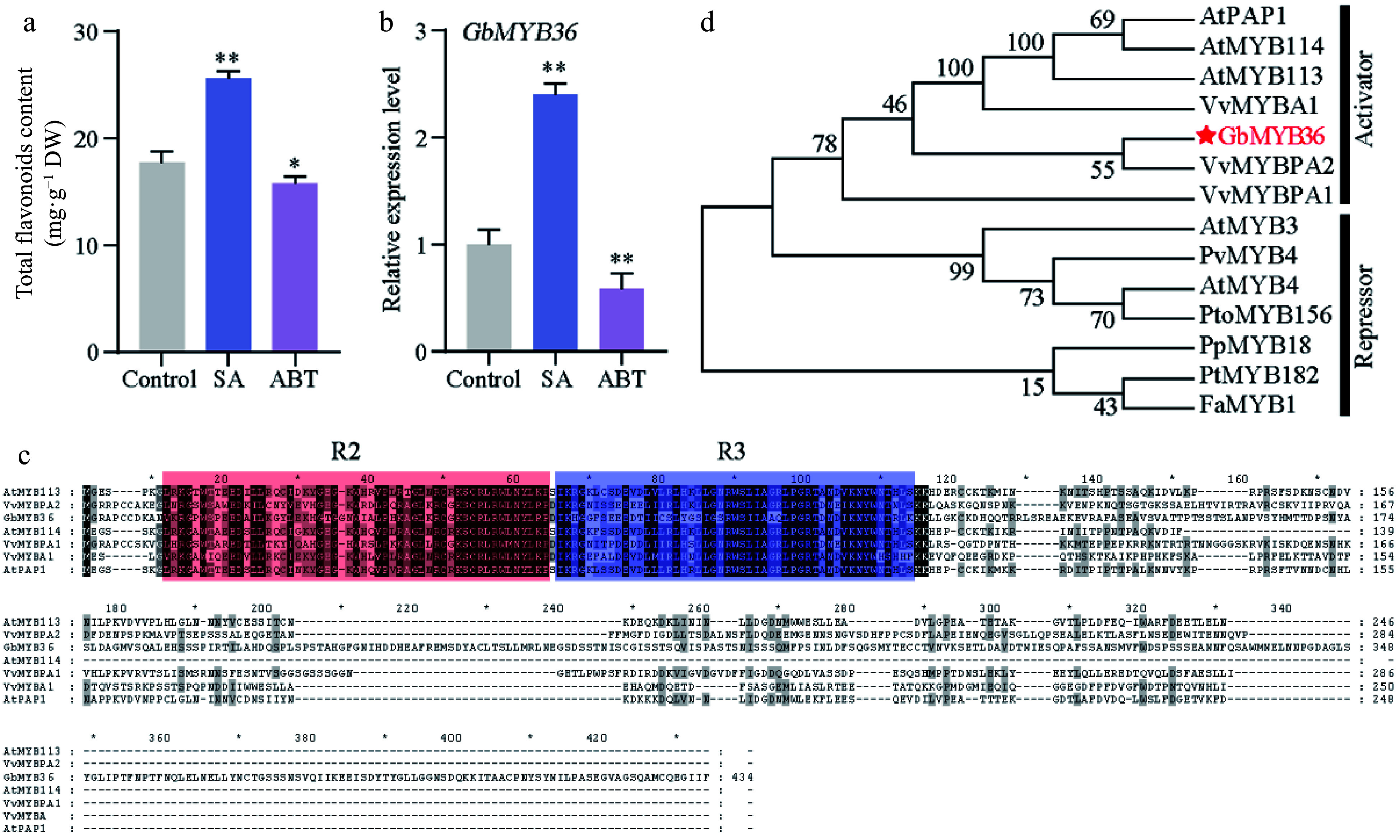
The characteristics of GbMYB36 transcription factor. (a) Total flavonoid content in SA- and ABT-treated leaves. (b) *GbMYB36* expression levels in SA- and ABT-treated leaves. (c) Phylogenetic analysis of the R2R3-MYBs in ginkgo and other plant species. (d) Amino acid sequence alignment of GbMYBs and other known MYB activators and repressors. The following GenBank accession numbers were used: *Arabidopsis thaliana* AtMYB4 (NP_195574), AtMYB3 (NP_564176), AtPAP1 (Q9FE25), AtMYB113 (Q9FNV9), AtMYB114 (Q9FNV8); *Vitis vinifera* VvMYBA1 (BAD18977), VvMYBPA1 (CAJ90831), VvMYBPA2 (ACK56131); *Prunus persica* PpMYB18 (ALO81021); *Populus tremula* × *Populus tremuloides* PtMYB182 (AJI76863); *Fragaria* × *ananassa* FaMYB1 (AAK84064); *Panicum virgatum* PvMYB4 (AEM17348); *Populus tomentosa* PtoMYB156 (AMY62793). Statistical significance: * *p* < 0.05; ** *p* < 0.01.

MYBs play major roles in flavonoid synthesis in plants^[11]^. To further functionally characterize the TFs in terms of their regulatory effects on SA-induced flavonoid synthesis, we analyzed their expression in the treated calli. The transcriptome results showed that the expression levels of most MYB genes were significantly higher in the calli exposed to SA than in the control (Supplemental Fig. S5). We further treated ginkgo seedlings with exogenous SA and ABT (SA inhibitor), and found that SA promoted the content of total flavonoids and the expression of *GbMYB36*. On the contrary, ABT treatment inhibited flavonoid synthesis and the *GbMYB36* expression level (Fig. 6a & b). The GbMYB36 were predicted as R2R3-MYB protein (Fig. 6c). We explored the phylogenetic relationships of GbMYB36 with known R2R3-MYB activators and repressors from different species. A phylogenetic tree divided these R2R3-MYB genes into two clades. GbMYB36 is within the clade of known flavonoid activators (e.g., AtPAP1, VvMYBPA2, and VvMYBA1) (Fig. 6d).

**Figure 7:**
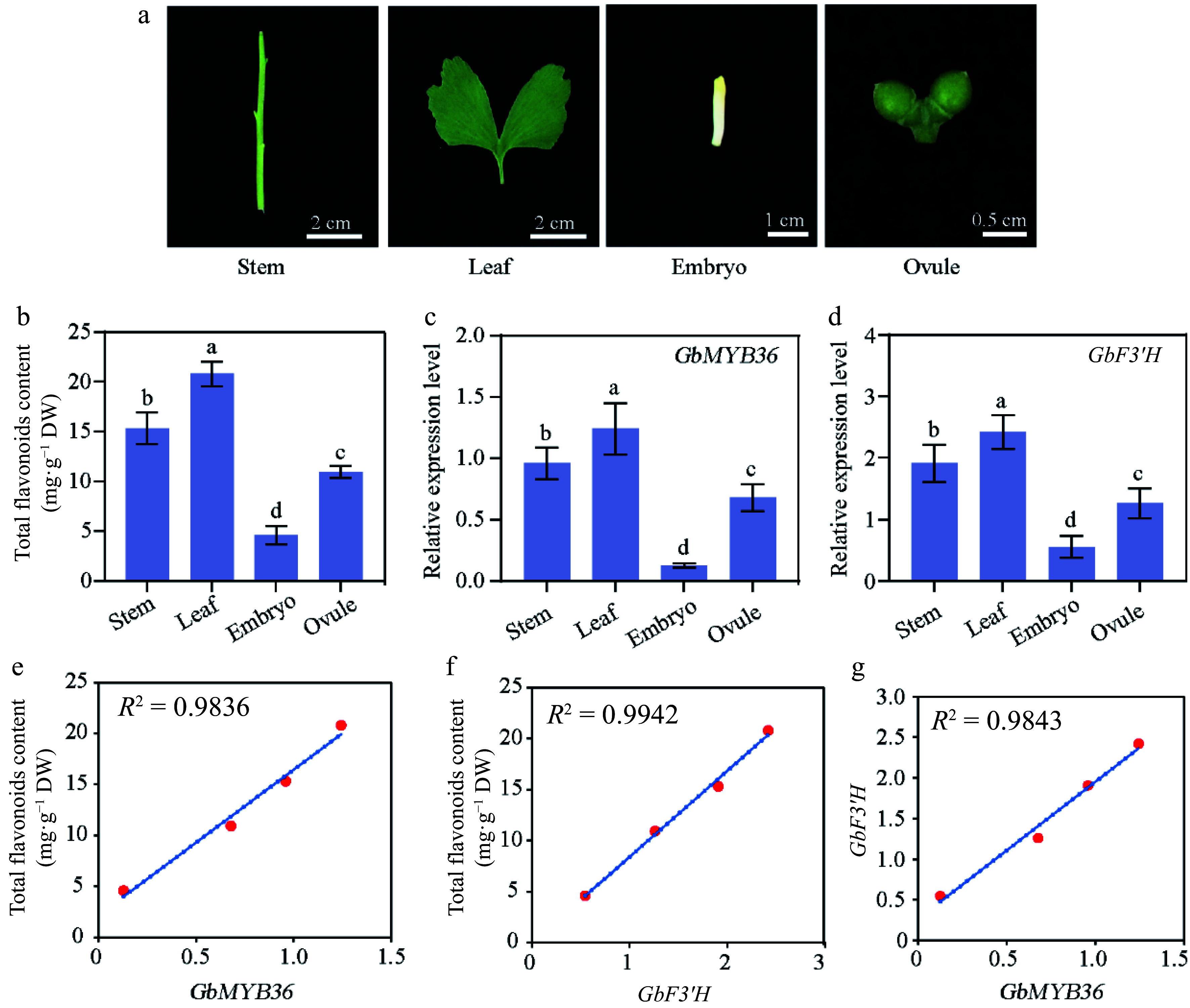
Flavonoid-related gene expression and flavonoid contents in different organs of *G. biloba*. (a) The different organs of ginkgo include the stem, leaf, embryo, and ovule tissues. (b) Flavonoid content within different organs. The expression of (c) *GbMYB36* and (d) *GbF3′H* genes in different organs. (e), (f) Correlation analysis of gene expression and flavonoid contents. (g) The correlation coefficient of the expression levels of *GbMYB36* with *GbF3′H*. Different letters indicate statistically significant differences (Tukey‘s test; *p* < 0.05).

To further determine whether *GbMYB36* expression was correlated with flavonoid accumulation, we analyzed the expressions of the *GbMYB36* gene and the key flavonoid biosynthesis gene, *GbF3′H*, in different organs (roots, stems, leaves, embryos, and ovules) (Fig. 7a). The expression levels of *GbMYB36* and *GbF3′H* were highest in leaves and lowest in embryos (Fig. 7c & d). The content of flavonoids was also highest in leaves, followed by stems, and lowest in embryos (Fig. 7b). Furthermore, correlation coefficients were calculated to assess the relationship between gene expression and flavonoid content. *GbMYB36* and *GbF3′H* were strongly correlated with the flavonoid content, with correlation coefficients of 0.98 and 0.99, respectively (Fig. 7e & f). We also found that the expression levels of *GbMYB36* and *GbF3′H* were positively correlated (R^2^ = 0.98) (Fig. 7g). Accordingly, we speculated that *GbMYB36* is involved in the regulation of flavonoid synthesis.

### Overexpression of GbMYB36 in ginkgo promoted *GbF3'H* transcription and flavonoid accumulation

**Figure 8:**
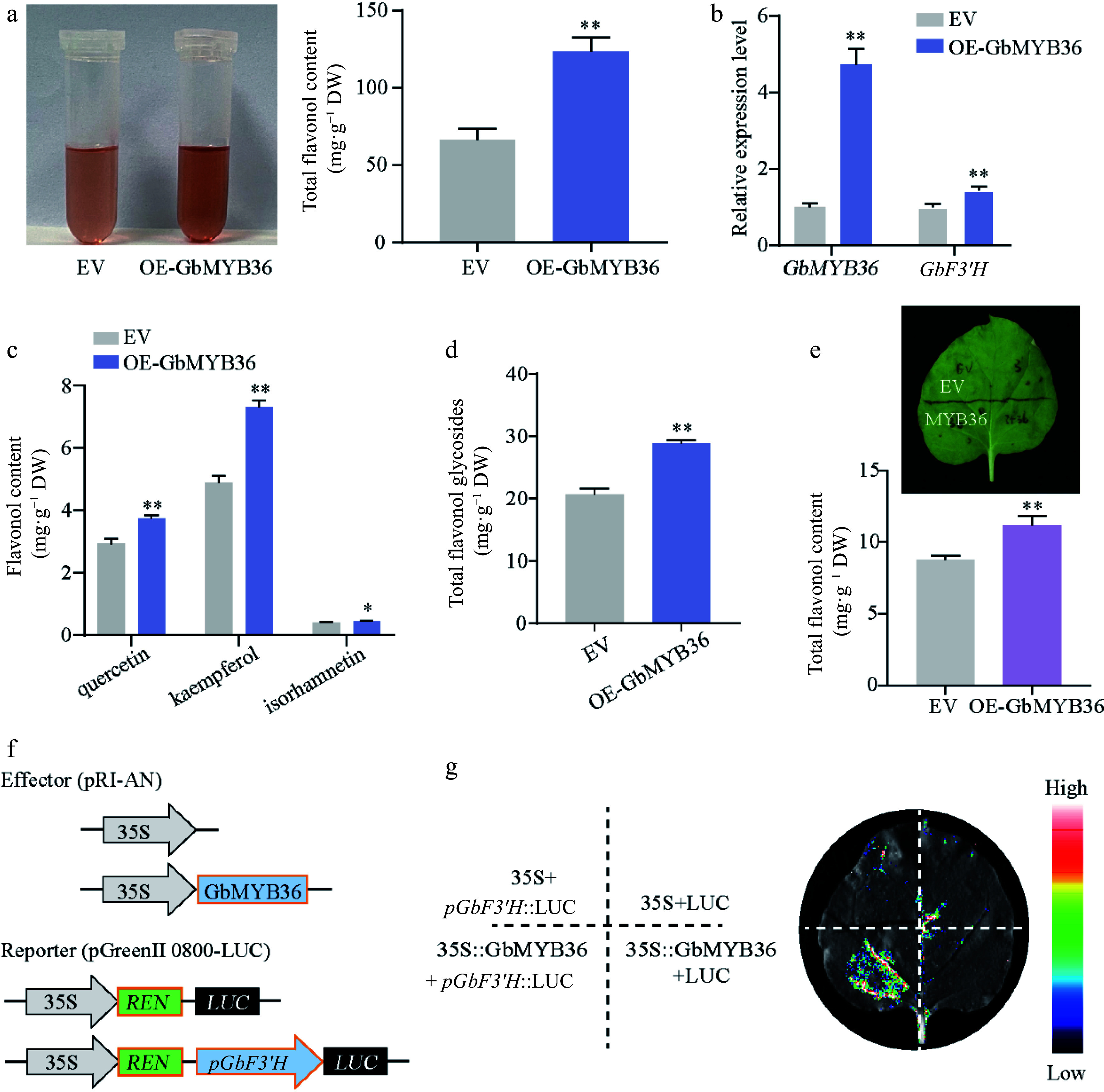
Functional analysis of *GbMYB36* gene. (a) Total flavonoid contents in the EV and OE-GbMYB36 calli. (b) Gene expression levels of *GbMYB36* and *GbF3′H* in transient overexpression calli. (c), (d) HPLC analyses of flavonol in the EV and OE-GbMYB36 calli. (d) Production of flavonoids by the transient overexpression of GbMYB36 in tobacco leaves. (f) Schematic diagrams show the reporter and effector vectors used for dual luciferase reporter (DLR) assays (g) DLR assays to identify the activation of GbMYB36 on *GbF3′H*. Statistical significance: * *p* < 0.05; ** *p* < 0.01.

To further clarify whether *GbMYB36* participate in the regulation of flavonoid synthesis, we constructed the recombinant plasmid carrying *35S::GbMYB36*, which were transformed into ginkgo calli. The content of total flavonoids in GbMYB36 overexpression (OE-GbMYB36) calli was increased by 85% compared to the empty vector-transformed (EV) calli (Fig. 8a). *GbMYB36* was more highly expressed in their overexpression calli, indicating successful transformation (Fig. 8b). Furthermore, we detected the flavonol contents in OE-GbMYB36 calli. Compared with the control calli, the quercetin, kaempferol, isorhamnetin and total flavonol glycosides contents in OE-GbMYB36 calli were dramatically increased by 27.78%, 42.52%, 11.59% and 39.92%, respectively (Supplemental Fig. S6; Fig 8c & d). Furthermore, transient overexpression in tobacco leaves indicated that infiltration of *GbMYB36* significantly increased flavonoid accumulation (by approximately 27.5%) (Fig. 8e). These results suggest that *GbMYB36* may play the key role in flavonoid synthesis.

We also measured the expression levels of the vital *GbF3′H* gene by qRT-PCR and found significantly higher *GbF3′H* expression in the OE-GbMYB36 calli than in the control (Fig. 8b). To investigate further how GbMYB36 specifically regulates *GbF3′H*, we performed a dual luciferase reporter (DLR) assay. The *GbF3′H* promoter fragment was fused to a firefly luciferase (LUC) reporter sequence (*pGbF3′H*::LUC) and co-transformed into *N. benthamiana* leaves with either 35S::GbMYB36 or an empty vector. The results revealed that *GbF3′H* promoter drives LUC expression weakly without GbMYB36, but co-expression of *pGbF3′H*::LUC with GbMYB36 led to a strong luciferase signal (Fig. 8f & g). These results indicate that GbMYB36 directly activates the expression of *GbF3′H* to modulate flavonoid accumulation.

## Discussion

### Tissue culture and optimum medium screening in *G. biloba*

Plants possess a biosynthesis machinery that can produce multitudinous active compounds. These compounds are important sources of pharmaceuticals, food additives, and other industrially significant products^[2,37]^. Plant tissue culture is considered an attractive alternative to traditional cultivation technologies because it offers a viable source of important secondary metabolites^[38]^. Additionally, bioreactor systems for the production of active bioactive compounds by plant organ culture has emerged as an efficient technology with possible commercial applications^[39]^. *G. biloba* leaves contain abundant active compounds, especially flavonoids, which have strong free radical scavenging activity and antioxidant capacities^[3]^. However, the high phenol content in ginkgo leaves resulted in serious browning of callus during tissue culture, which hinders the establishment of a high efficiency tissue culture system for ginkgo.

Several previous studies found that AC, VC, and PVP effectively inhibit tissue browning in various plants^[40]^. In this study, we evaluated these potential anti-browning agents for their inhibitory effects on the callus subculture of *G. biloba* to identify the best anti-browning agent. Assays to determine the browning rate demonstrated that VC was considerably better than PVP and AC as an anti-browning reagent during callus culturing of *G. biloba* leaves. Moreover, VC and AC effectively suppressed the activity of PPO, which can interact with phenolic compounds, resulting in browning reactions^[36]^. Consistent with our results, it has been reported that VC has a positive effect on anti-browning and anti-PPO activity in sprouts^[40]^. However, VC exhibited only a marginal inhibitory effect on browning and PPO activity in potato^[41]^. We attribute the reason for the differences in anti-browning effects and PPO activity to the varying responses of different species to VC. Overall, VC can significantly suppress browning and PPO activity during the callus subculture of *G. biloba*, suggested that VC at low concentration was effective browning antagonist in tissue culture of ginkgo. Based on the results, we established the optimal induction and subculture media of ginkgo callus.

### Exogenous SA treatment significantly promotes *F3'H* expression and flavonoid accumulation in *G. biloba*

Although plant tissue culture is an efficient technology for several active compounds, there exists a need for efficient methods to improve the content of active ingredients^[42]^. An efficient strategy to promote the production of active compounds from *in vitro* cultures is to add elicitors that trigger the formation of secondary metabolites^[21]^. SA is a well-known elicitor and plays a substantial role in the induction of secondary metabolites^[43]^. Previous studies have reported a higher flavonoids content in SA-treated *Fagonia indica* callus compared with the control^[44]^. In our study, the exogenous application of SA promoted flavonol production in ginkgo callus, including quercetin, kaempferol and isorhamnetin, confirming SA-induced flavonoid biosynthesis in *G. biloba*.

The regulation of the production of some compounds can be achieved by understanding the genes involved in the pivotal metabolic pathways^[2]^. Here, we used transcriptomic analysis to comprehensively identify the effects of SA on the expression of genes participating in flavonoid synthesis. Almost all genes related to flavonoid synthesis were upregulated in SA-treated callus. Particularly, among them, the transcript abundance of *GbF3′H* increased dramatically. F3′H is a cytochrome P450 monooxygenase that catalyzes the hydroxylation of 3′-position of flavonoid B-ring to the 3′,4′-hydroxylated state during flavonoid biosynthesis^[45,46]^. In *A. thaliana*, the F3′H enzyme can convert dihydrokaempferol to dihydroquercetin, a precursor for the synthesis of quercetin^[45,47]^. To date, the F3′H enzyme has been cloned and functionally analyzed in various species^[48]^. The deletion mutation of the *F3′H* genes affects the pigmentation of the seeds in Arabidopsis and soybean^[45,49,50]^. In tartary buckwheat, overexpression of *F3′H* resulted in a significant increase in anthocyanin contents^[51]^. Furthermore, ectopic expression of apple *F3′H* genes contributes to quercetin accumulation^[52]^. In this study, we used genetic transformation to demonstrate that *GbF3′H* is a flavonoid-specific regulator and its transient expression led to the accumulation of quercetin, kaempferol, and isorhamnetin in ginkgo calli.

### *GbMYB36* activates the transcription of *F3'H* to promote flavonoid accumulation

Most flavonoid biosynthetic pathway genes have been identified in many plants, and genetic engineering of specific genes has been used to improve the production of flavonoid compounds^[21]^. Additionally, there is growing evidence that the expression levels of flavonoid-related genes are predominantly regulated by several transcription factors directly binding to their promoters^[11]^. Certain studies report that the MBW (MYB, bHLH, and WD40) complex can activate the structural genes of flavonoid pathway, resulting in the flavonoids accumulation^[13]^. Various MYB TFs are involved in flavonoid accumulation, especially R2R3‐MYB TFs^[14]^. After SA treatment, MYB TFs are activated, thereby enhancing the flavonoid accumulation in poplar^[23]^. In this study, through the RNA-seq analysis, we found the expression of several MYB members were significantly upregulated after SA treatment. In particular, we identified a new R2R3‐MYB TF, GbMYB36, which strongly responded to SA treatment. GbMYB36 was grouped phylogenetically within known positive regulators of flavonoid biosynthesis and its expression was highly correlated with flavonoid-specific *GbF3′H* expression. Meanwhile, overexpressing *GbMYB36* in ginkgo significantly promoted the expression of *GbF3′H* gene as well as the flavonoid content. In addition, a LUC assay suggested that GbMYB36 can directly activate the expression of *GbF3′H*. These findings suggest that *GbMYB36* might be a key regulator of SA‐induced flavonoid biosynthesis, which can activate the expression of the flavonoid biosynthesis gene *GbF3′H* to promote flavonoid accumulation after SA stimulation.

## Conclusions

In this work, we screened the optimal induction and subculture media for ginkgo calli. On this basis, SA treatment remarkably increased flavonoid contents in ginkgo calli, particularly for quercetin, kaempferol, isorhamnetin, and total flavonol glycosides. The transcriptomic results indicated *GbF3′H* gene related to flavanone 3′-hydroxylase in response to SA. Further genetic transformation demonstrated that *GbF3′H* is a flavonoid-specific regulator and its transient expression led to the accumulation of quercetin, kaempferol, isorhamnetin and total flavonol glycosides. In addition, transcriptome and qPCR analysis revealed that SA markedly induced *GbMYB36* expression. In particular, GbMYB36 directly activates the *GbF3′H* expression to modulate flavonoid accumulation, suggesting the important role of *GbMYB36* in SA-induced flavonoid biosynthesis.

## SUPPLEMENTARY DATA

Supplementary data to this article can be found online.
