# Supplementary material for: SA-responsive transcription factor GbMYB36 promotes flavonol accumulation in Ginkgo biloba
Source: For Res (Fayettev). 2023 Aug 10;3:19. doi: 10.48130/FR-2023-0019 (PMC11524253; doi:10.48130/FR-2023-0019)
Supplement: Supplementary file 1 — Supplementary data to this article can be found online. [file FR-2023-0019-S1.zip › 10.48130_FR-2023-0019-Suppl-FigureS1.docx]

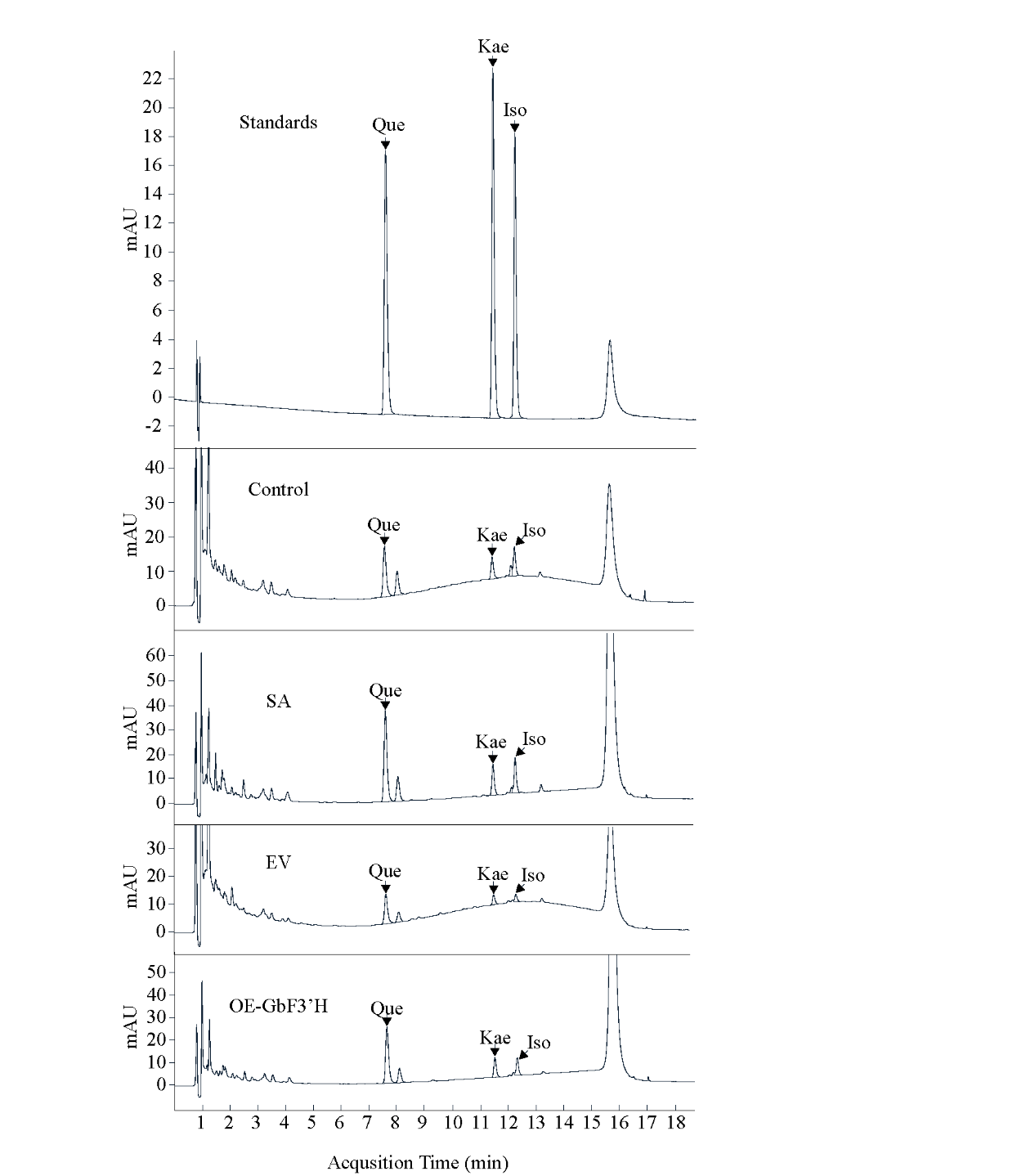


**Fig. S1** Identification of flavonol in the flavonoid extract. The flavonoid compounds in the extract were separated by HPLC and identified by comparison with the retention time of the standards. Three abundant flavonol glycosides compounds (quercetin, Que; kaempferol, Kae; isorhamnetin, Iso) were detected
