# Supplementary material for: SA-responsive transcription factor GbMYB36 promotes flavonol accumulation in Ginkgo biloba
Source: For Res (Fayettev). 2023 Aug 10;3:19. doi: 10.48130/FR-2023-0019 (PMC11524253; doi:10.48130/FR-2023-0019)
Supplement: Supplementary file 1 — Supplementary data to this article can be found online. [file FR-2023-0019-S1.zip › 10.48130_FR-2023-0019-Suppl-FigureS4.docx]

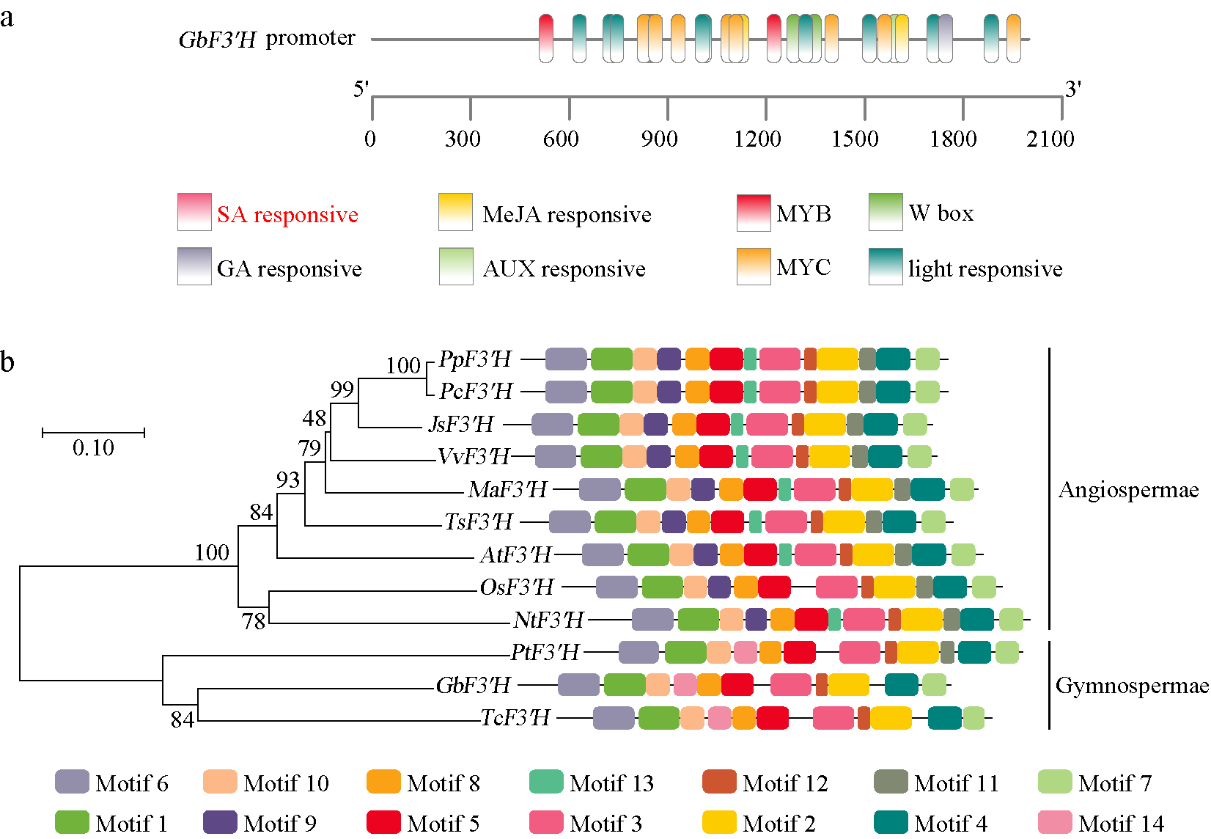


**Fig. S4** The characteristics of *GbF3′H*. (A) Identification of cis-acting elements of *GbF3′H* promoter. (B) Phylogenetic tree analysis of GbF3′H proteins from other species (*Triadica* *sebifera*, *Oryza* *sativa*, *Arabidopsis* *thaliana*, *Vitis* *vinifera*, *Juglans* *sigillata*, *Prunus* *persica*, *Narcissus* *tazetta*, *Prunus* *cerasifera*, *Morus* *alba*, *Taxus* *chinensis*, and *Pinus* *taeda*).
