# Supplementary material for: SA-responsive transcription factor GbMYB36 promotes flavonol accumulation in Ginkgo biloba
Source: For Res (Fayettev). 2023 Aug 10;3:19. doi: 10.48130/FR-2023-0019 (PMC11524253; doi:10.48130/FR-2023-0019)
Supplement: Supplementary file 1 — Supplementary data to this article can be found online. [file FR-2023-0019-S1.zip › 10.48130_FR-2023-0019-Suppl-TableS1.docx]

**Table S1. Composition of the media used for the induction of callus**

| **Medium** | **2,4-D**  **(mg·L^-1^)** | **NAA**  **(mg·L^-1^)** | **KT**  **(mg·L^-1^)** |
| --- | --- | --- | --- |
| M1 | 2.0 | 0 | 1.0 |
| M2 | 2.0 | 0 | 2.0 |
| M3 | 2.0 | 0 | 4.0 |
| M4 | 0 | 1.0 | 2.0 |
| M5  M6  M7  M8  M9 | 0  0  1.0  2.0  4.0 | 2.0  4.0  2.0  2.0  2.0 | 2.0  2.0  0  0  0 |
