# Supplementary material for: SA-responsive transcription factor GbMYB36 promotes flavonol accumulation in Ginkgo biloba
Source: For Res (Fayettev). 2023 Aug 10;3:19. doi: 10.48130/FR-2023-0019 (PMC11524253; doi:10.48130/FR-2023-0019)
Supplement: Supplementary file 1 — Supplementary data to this article can be found online. [file FR-2023-0019-S1.zip › 10.48130_FR-2023-0019-Suppl-TableS2.docx]

**Table S2.** **Statistics of leaf callus induction rate under different treatments**

| **Medium** | **induction rate /%**  **(after 15 d)** | **induction rate /%**  **(after 30 d)** | **induction rate /%**  **(after 45 d)** |
| --- | --- | --- | --- |
| M1 | 23.33 ± 4.71^cd^ | 36.67 ± 4.71^cd^ | 43.33 ± 4.71^b^ |
| M2 | 26.67 ± 4.71^bcd^ | 40 ± 4.71^bcd^ | 50 ± 8.17^b^ |
| M3 | 16.67 ± 12.5^d^ | 26.67 ± 12.5^d^ | 36.67 ± 4.71^b^ |
| M4 | 16.67 ± 4.71^d^ | 43.33 ± 9.43^abcd^ | 50 ± 8.17^b^ |
| M5 | 33.33 ± 4.71^bc^ | 43.33 ± 4.71^abcd^ | 46.67 ± 4.71^b^ |
| M6 | 50 ± 8.17^a^ | 56.67 ± 9.43^ab^ | 66.67 ± 9.43^a^ |
| M7 | 36.67 ± 4.71^abc^ | 46.67 ± 4.71^abc^ | 63.33 ± 4.71^a^ |
| M8 | 26.67 ± 4.71^bcd^ | 40 ± 8.17^bcd^ | 43.33 ± 4.71^b^ |
| M9 | 40 ± 8.17^ab^ | 60 ± 0^a^ | 63.33 ± 4.71^a^ |
