# Supplementary material for: SA-responsive transcription factor GbMYB36 promotes flavonol accumulation in Ginkgo biloba
Source: For Res (Fayettev). 2023 Aug 10;3:19. doi: 10.48130/FR-2023-0019 (PMC11524253; doi:10.48130/FR-2023-0019)
Supplement: Supplementary file 1 — Supplementary data to this article can be found online. [file FR-2023-0019-S1.zip › 10.48130_FR-2023-0019-Suppl-TableS3.docx]

**Table S3. Primers used in this work.**

| **Name** | **Sequence** | **Application** |
| --- | --- | --- |
| ***Gb24534_F*** | CATGATGGCTGCTGGATCGGATAC | RT-qPCR |
| ***Gb24534_R*** | CGACTCTTGCCCACCACTGATTC | RT-qPCR |
| ***Gb04545_F*** | GGAATGACAGAACTGATGAGGAACC | RT-qPCR |
| ***Gb04545_R*** | ACGCACAGCAGGTAGTCCAG | RT-qPCR |
| ***Gb32735_F*** | ACTGTGGTGGCGTCTTCTCC | RT-qPCR |
| ***Gb32735_R*** | AAAGTCATAGGCGGTGTATTTCCC | RT-qPCR |
| ***Gb11310_F*** | TACAGGGCAGCAAGACATCAAATG | RT-qPCR |
| ***Gb11310_R*** | AGATTCTGGAGAGTTCGGTGAGG | RT-qPCR |
| ***GbMYB36_F*** | CAGGTCTGAAGCGATGCGGTAAG | RT-qPCR |
| ***GbMYB36_R*** | GCTTCCAATGCTGCCGTAGAGG | RT-qPCR |
| ***actin_F*** | CTGCCAAGGCTGTAGGTAAGG | RT-qPCR |
| ***actin_R*** | TCAGATTCCTCCTTGATGGCG | RT-qPCR |
| ***Gb24534_F*** | gaccccgggggtaccggatccATGTCTTCTCTGGAGTTCAATATGCC | Construct *PRI-GbF3′H* |
| ***Gb24534_R*** | tttacccatgaattcggatccAAGGCGGTTCATGTCTAACCTTG | Construct *PRI-GbF3′H* |
| ***GbMYB36_F*** | gaccccgggggtaccggatccATGGGCAGGGCTCCTTGC | Construct *PRI-GbMYB36* |
| ***GbMYB36_R*** | tttacccatgaattcggatccAAAGATTATTCCCTCTTGACACATTG | Construct *PRI-GbMYB36* |
| ***pGbF3′H _F*** | ttcctgcagcccgggggatccCACTAAATGTAGTAGTGGAGTGAGTGTATG | LUC |
| ***pGbF3′H _R*** | tgtttttggcgtcttccatggTATGCTCTGCAATAGTAGCTGTCTCTC | LUC |
