# Supplementary figures and images for: SA-responsive transcription factor GbMYB36 promotes flavonol accumulation in Ginkgo biloba
Source: For Res (Fayettev). 2023 Aug 10;3:19. doi: 10.48130/FR-2023-0019 (PMC11524253; doi:10.48130/FR-2023-0019)

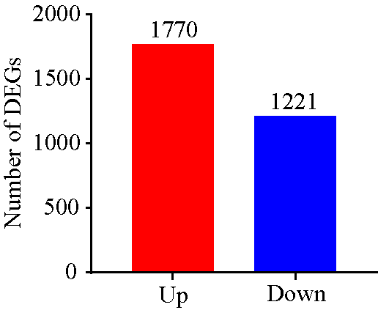


**Fig. S****2** Differentially expressed genes (DEGs) analysis of calli between SA treatment and control.

Supplement: Supplementary file 1 — Supplementary data to this article can be found online. [file FR-2023-0019-S1.zip › 10.48130_FR-2023-0019-Suppl-FigureS2.docx]

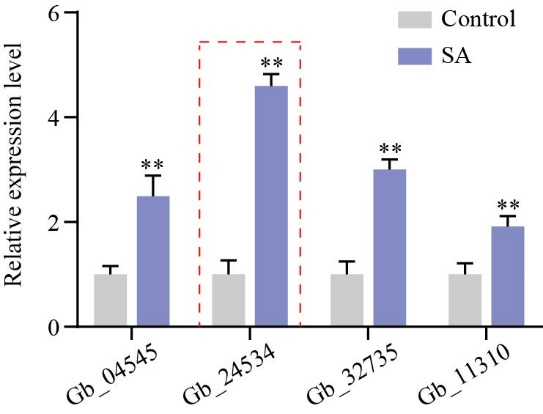


**Fig. S3** The expression levels of four vital *F3’H* (*Gb_04545*, *Gb_24534*, *Gb_32735* and *Gb_11310*) genes in SA treated calli and control.

Supplement: Supplementary file 1 — Supplementary data to this article can be found online. [file FR-2023-0019-S1.zip › 10.48130_FR-2023-0019-Suppl-FigureS3.docx]

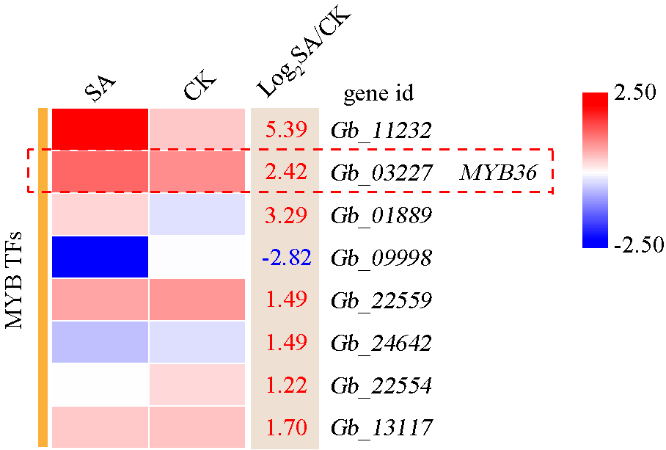


**Fig. S5** Expression profiles of MYB transcripts after SA treatment and in the control.

Supplement: Supplementary file 1 — Supplementary data to this article can be found online. [file FR-2023-0019-S1.zip › 10.48130_FR-2023-0019-Suppl-FigureS5.docx]

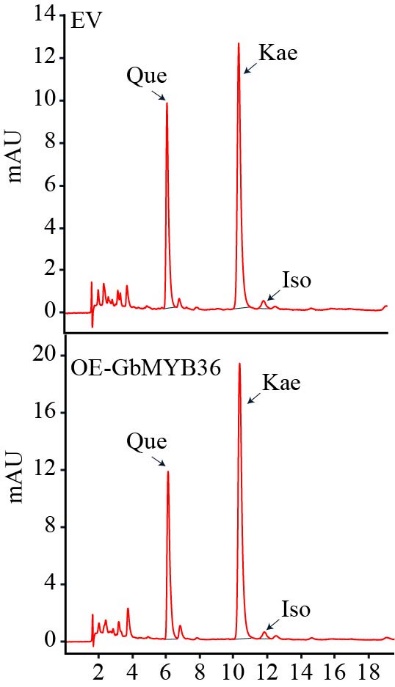


**Fig. S6** Identification of flavonol in the EV and OE-GbMYB36 calli.

Supplement: Supplementary file 1 — Supplementary data to this article can be found online. [file FR-2023-0019-S1.zip › 10.48130_FR-2023-0019-Suppl-FigureS6.docx]
